# Data on making uniform lignin building blocks via in-situ real-time monitoring of hydroxyethyl modification

**DOI:** 10.1016/j.dib.2020.106512

**Published:** 2020-11-10

**Authors:** Li-Yang Liu, Kim Bessler, Siwei Chen, Mijung Cho, Qi Hua, Scott Renneckar

**Affiliations:** Department of Wood Science, The University of British Columbia, 2424 Main Mall, Vancouver, British Columbia, Canada

**Keywords:** lignin, Real-time monitoring, Hydroxyethyl modification, Carbon storage, Building blocks, Bioplastics

## Abstract

In this work, a lab-designed apparatus was developed to collect and record the CO_2_ amount during the hydroxyethyl modification of lignin. We presented the CO_2_ volume amount and the production rate under different reaction conditions (80 – 120 °C and 2 – 6 hrs). Nuclear magnetic resonance spectroscopy was performed to analyze the chemical structure of the hydroxyethyl lignin corresponding with different amounts of CO_2_ that evolved during the reaction. The aliphatic hydroxyl, aromatic hydroxyl, and carboxylic acid groups were analyzed and tabulated. The acetylated hydroxyethyl lignin samples were characterized by ^13^C NMR to obtain the aliphatic hydroxyl (primary and secondary), phenol (ortho substituted and ortho-free), hydroxyethyl, methoxy, and aromatic hydrogen groups semi-quantitatively. Fourier-transform infrared (FTIR) spectroscopy was adopted to analyze the surface functional groups including alkyl aryl ether bond, carboxylic acid groups, and aromatic hydroxyl groups. Gel permeation chromatography combined with a multi-angle light scattering detector and differential refractive index detector were used to obtain the molar mass of lignin before and after the modification.

## Specifications Table

**Table:** 

Subject	Process Chemistry and Technology
Specific subject area	This data set covers a greener chemical modification procedure to obtain more uniform aromatic polymer resources using in-situ real-time monitoring of the reaction
Type of data	Table, Image, Graph, and Fig.
How data were acquired	In-situ Real-time monitoring: - The hydroxyethyl reactions were performed in a 50 mL round bottom flask by mixing 2.5 g dried lignin powders with 8 g ethylene carbonate and 0.4 g sodium carbonate (Na_2_CO_3_). - A water displacement method was used to measure the volume of CO_2_ during the hydroxyethyl reaction [1]. The equipment was displayed in Fig. 1. The volume of CO_2_ during the hydroxyethyl reaction was recorded every 10 - 15 min. - The hydroxyethyl reaction was then quenched by adding 200 mL cold water, stirred overnight, washed and filtered by another 3 × 200 mL distilled water, dried by lypholization, and kept in the vacuum oven (50 °C) before characterization.
	Nuclear magnetic resonance spectrum: - Solution state NMR (^13^C, ^31^P, and ^1^H) were obtained using 300 MHz NMR machine equipped with BBO probe. (Bruker Avance, Bruker Corp., US). - ^31^P NMR samples preparation: A solution was prepared by mixing pyridine and CDCl_3_ in a ratio 1.6/1 v/v. The pyridine was protected from moisture with molecular sieves. The relaxation reagent and internal standard were prepared by dissolving the chromium(III) acetylacetonate and N-hydroxy-5-norbornene-2,3-dicarboximide into the solution with a concentration of 5.6 mg/mL and 10.0 mg/mL, respectively. An exact amount of 20 mg dried lignin powder was then dissolved in 400 μL above solution, followed with the addition of 100 μL internal standard solution, 40 μL relaxation reagent solution, and 50 μl 2-Chloro-4,4,5,5-tetramethyl-1,2,3-dioxaphospholane (TMDP) [2], [3], [4]. - ^13^C NMR sample preparation: approximately 150 mg of lignin or acetylated lignin was dissolved into 450 μl DMSO-_d6_, followed by the addition of 60 μl of a solution of chromium(III) acetylacetonate (50 mg/mL) in DMSO-d6 as relaxation reagent and 15 mg of trioxane as internal standard [4,5]. - These lignin solutions were thoroughly mixed until no solid was left in the solution and transferred into a 5 mm NMR tube for immediate analysis. - The obtained spectra were processed using the Topspin 3.6.1 software including Fourier transform, phase correction, baseline change, and calibration.
	Gel permeation chromatography: - Agilent 1100 HPLC system including a guard column (PL PolarGel L) and two GPC column (PolarGel L and M, Agilent). - The fractionated samples were then analyzed using a multi-angle light scattering detector (MALS, Wyatt Corp. US) and optilab T-rEX differential refractive index detector (dRI, Wyatt Corp. US). - The obtained molecular mass traces were collected and analyzed using Astra 6.1 software including conventional calibration analysis, Zimm plots, and dn/dc analysis.
Data format	Raw and Analyzed
Parameters for data collection	In-situ Real-time monitoring: - The reaction temperature of oil bath was set from 80 – 120 °C with time length from 0 – 6 hrs. - The CO_2_ amounts were collected and measured under atmospheric pressure (Vancouver, 102.3 – 101.3 kPa) and room temperature (20 – 25 °C). Nuclear magnetic resonance analysis: - Probe temperature: 25 °C. - *^31^P NMR:* an inverse-gated decoupling pulse sequence was employed with parameters: relaxation delay 5 s, acquisition time1.4 s, pulse length 6 μs, 90° pulse width, and number scan 800. The chemical shift of each phosphitylation product was calibrated with a product of TMDP with water (132.2 ppm) [6]. - *^13^C NMR:* an inverse-gated decoupling sequence was applied with parameters: relaxation delay 2 s, acquisition time 1.4 s, pulse length 8.15 μs, 90° pulse angle, and scan numbers 20,000. Signals were calibrated using DMSO as a reference (δ = 39.5 ppm). Attenuated total reflectance – Fourier transform infrared analysis - The lignin powders were analyzed using INVENIO ATR FT-IR (Bruker Corp.) with parameters: 4000 – 400 cm^−1^, scan number 64, and resolution 4 cm^−1^. Molar mass analysis: - Sample preparation: 10 mg acetylated lignin was dissolved in 1 mL DMSO/LiBr (0.5% w/v) at room temperature overnight. Before the analysis, samples were filtered using 0.45 µm PTFE filters. - Agilent 1100 HPLC system: flow rate 0.5 mL/min, column temperature 35°C - MALS detector: wavelength 785 nm at 35°C. - dRI detector: 35°C.
Data source location	Primary data sources were collected and located in the Advanced Renewable Materials Lab at The University of British Columbia, Forest Sciences Centre, Vancouver, Canada, Latitude: 49.2606° N, longitude: 123.2460° W
Data accessibility	NMR and GPC Data are available on Mendeley Dataset Repository: Data identification number: http://dx.doi.org/10.17632/9846nvt56s.1 Data URL: https://data.mendeley.com/datasets/9846nvt56s/1
Related research article	Li-Yang Liu, Kim Bessler, Siwei Chen, Mijung Cho, Qi Hua, Scott Renneckar, In-situ Real-time Monitoring of Hydroxyethyl Modification in Obtaining Uniform Lignin Derivatives. Eur. Polym. J. In press. DOI: https://doi.org/10.1016/j.eurpolymj.2020.110082

## Value of the Data

• This work provided a simple in-situ real-time monitoring method to obtain more uniform hydroxyethyl lignin.
• Researchers working on the modification of technical lignin resources will benefit from these data to reduce the workload on the optimization of reaction conditions, and enhance the reproducibility of modification, the quality of hydroxyethyl lignin, and overall economic efficiency.
• The lab-based CO_2_ measuring equipment and NMR analysis protocol can be adopted by other researchers working on the characterization and modification of lignin resources.
• The obtained hydroxyethyl lignin resources as building blocks with higher uniformity can be used to prepare materials, including but not limited to, polyurethane foams, polyesters, and coating materials.

## 1 Data Description

**Fig. 1:**
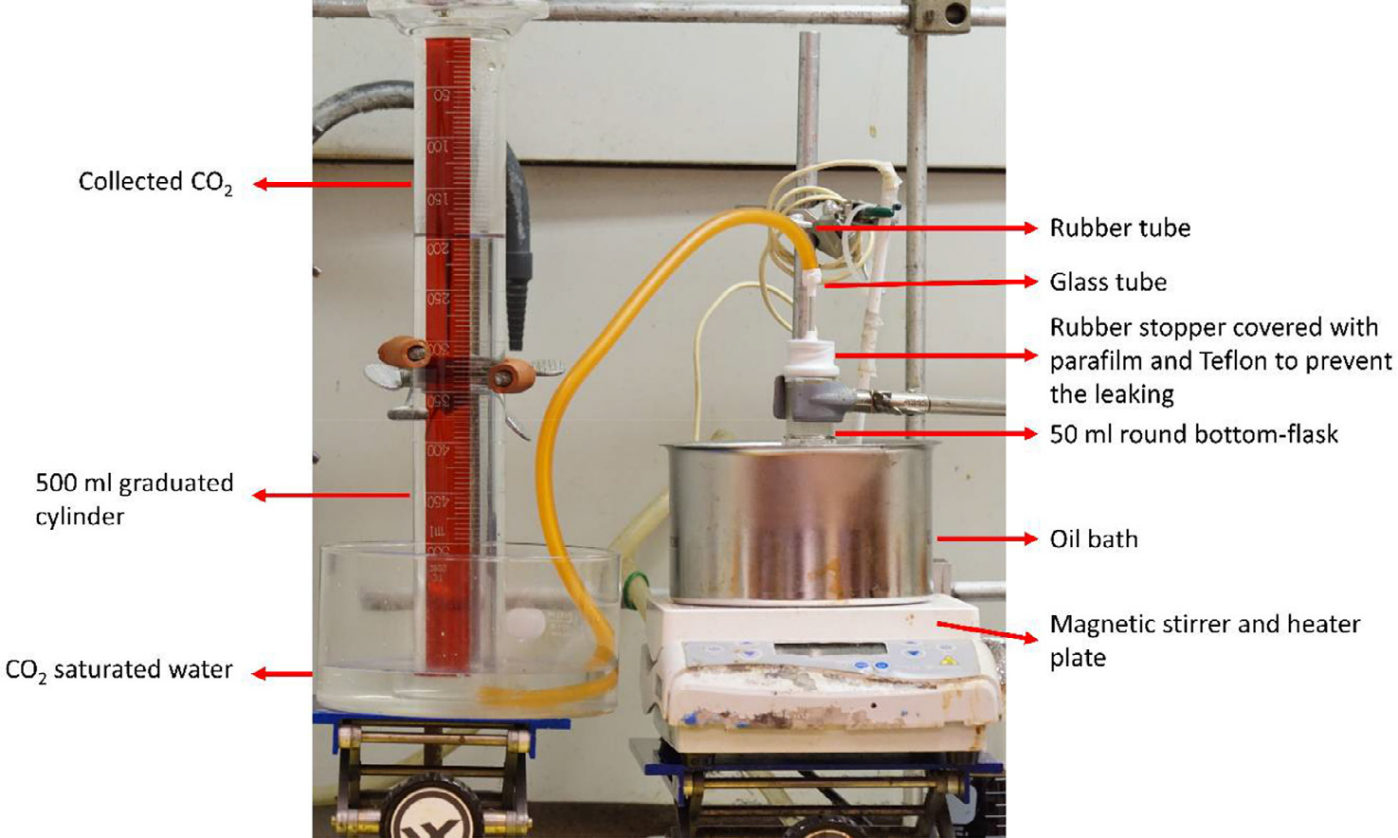
Lab-designed equipment for collecting and measuring CO_2_. The simple and accurate water displacement method was developed in the 18th-century [9] and is still widely used to record the generated gas, such as oxygen and hydrogen, during chemical reactions [10,11]. Carbon dioxide has a solubility of 1.45 g/L in aqueous water, so an excessive amount of CO_2_ was dissolved into the water to ensure the saturated conditions. All the connecting ports were carefully sealed to minimize the potential leaking. The reactor was pre-purged with a nitrogen atmosphere to dry the sample and prevent the backflow of water vapor causing the degradation of ethylene carbonate. The pressure of collected gas in the cylinder was equal to the atmospheric pressure (Vancouver, BC). The water partial vapor pressure is 3169.9 Pa with approximately 3% changes on the pressure of collected gas. It is worth to mention that the impacts from the buoyancy pressure of the water in the cylinder, and the slow leakage of CO_2_ gas for extended time periods (>6 hrs) may slightly impact the gas pressure in the cylinder as well. Here, we did not consider about these small changes to the volume.

**Fig. 2:**
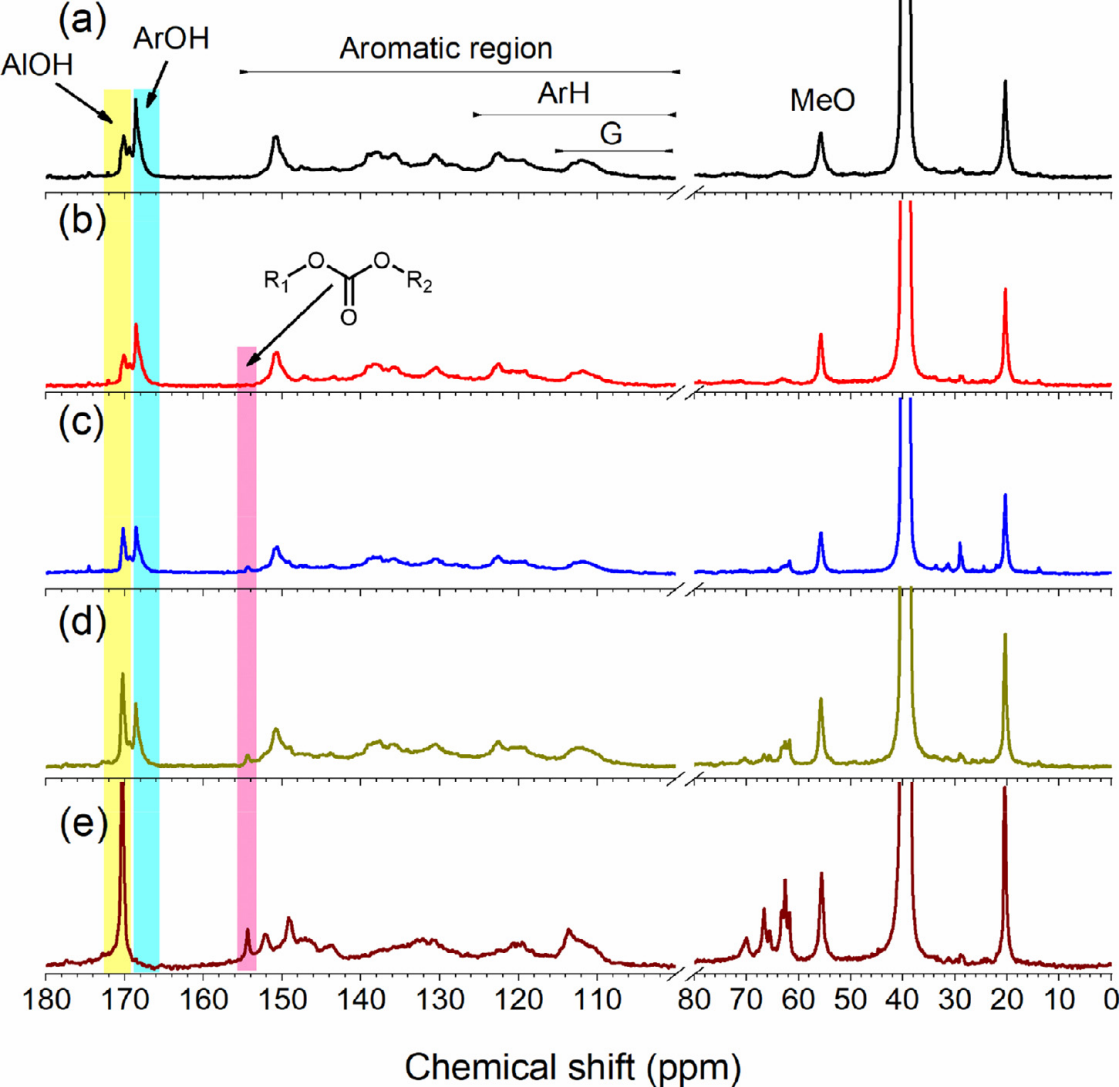
^13^C NMR spectra of hydroxyethyl lignin with a different amount of evolved CO_2_; (a) unmodified SKL; (b) CO_2_=10 mL/g; (c) CO_2_=32 mL/g; (d) CO_2_=54 mL/g; (e) CO_2_=86 mL/g; the carbon signal from the aromatic region (155-100 ppm) can be used as an internal standard. The relative concentration of functional groups is presented per 100 aromatic units. This was achieved by integrating the aromatic regions (100-155 pm) and setting this to a value 600 (100 aromatic ring), then all chemical groups would be expressed based on this value.

**Fig. 3:**
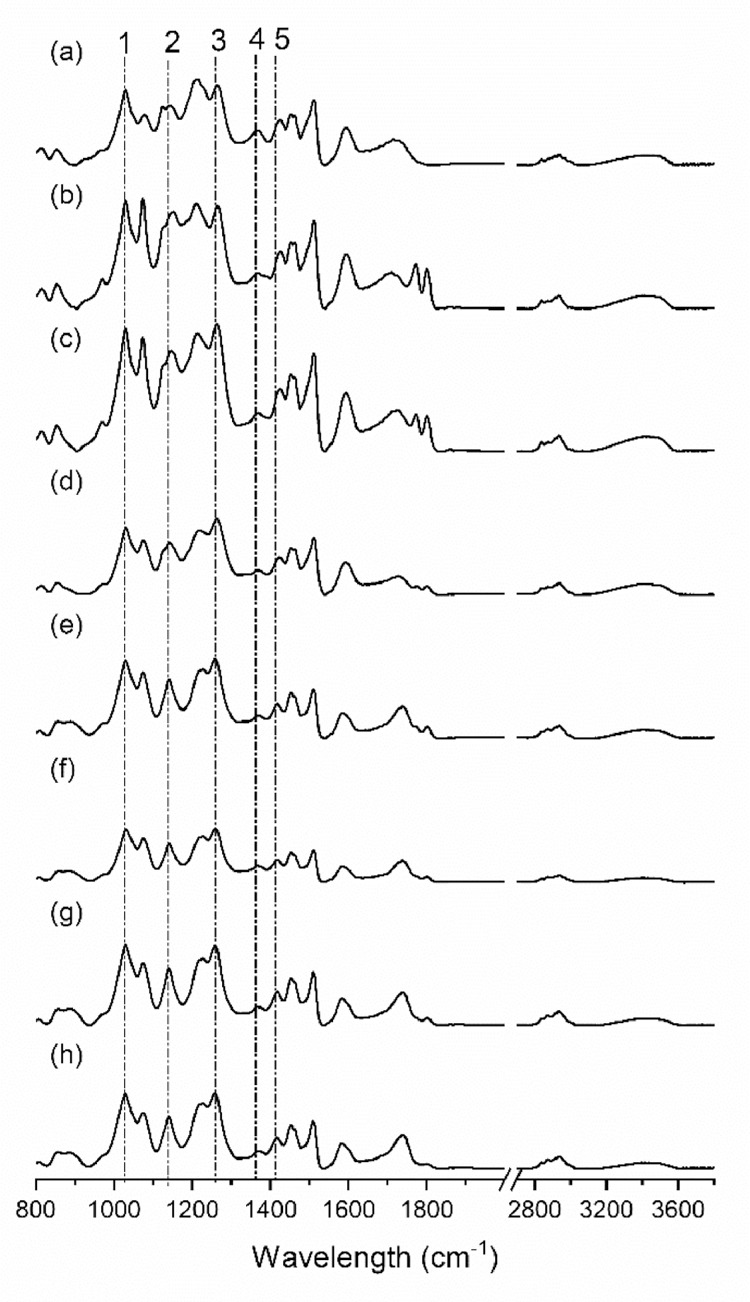
FT-IR spectra of hydroxyethyl lignin based on different amounts of evolved CO_2_; (a) unmodified SKL; (b) CO_2_=10 mL/g; (c) CO_2_=32 mL/g; (d) CO_2_=54 mL/g; (e) CO_2_=60 mL/g; (F) CO_2_=78 mL/g; (g) CO_2_=86 mL/g; (e) CO_2_=97 mL/g. alkyl aryl ether (P1, 1030cm^−1^ and P3, 1256 cm^−1^), primary alcohol (P2 1140 cm^−1^), phenol (p4, 1367 cm^−1^), and CH in plane (P5, 1418 cm^−1^).

In this study, Fig. 1 showed lab-designed equipment for collecting and measuring the CO_2_ amount during the hydroxyethyl modification of lignin. The in-situ real-time monitoring data, including the CO_2_ amount and CO_2_ production rate, were tabulated in Table 1. With different amounts of CO_2_, the modified lignin was analyzed by ^31^P and ^13^C NMR quantitatively based on the previous methods, while the original data was uploaded to the Mendeley Data [4,^5^,^7^,8]. Fig. 2 showed the ^13^C NMR spectra of modified lignin. The analyzed results were displayed in Tables 2 and 3. FT - IR spectra (Fig. 3) showed the chemical functional groups of lignin. The molar mass of unmodified and modified lignin samples were analyzed by the ASTRA software and tabulated in Table 4 [7]. The molecular weight traces were based on the MALS detector in our related research article [8].

## 2 Experimental Design, Materials and Methods

### 2.1 Materials and chemicals

**Table:** 

Lignin and chemicals	Brand	Note
Lignin resources	Amallin A^TM^ Kraft lignin	The obtained lignin resource has a pH = 4.2 at 15% solid contents. This resource was first washed with distilled water until the pH reached 5 - 6 before drying in the freeze dryer.
Ethylene carbonate	Alfa Aesar	Before using, this chemical was dried in the 40°C vacum oven for overnight at least
Deuterium chloroform	Cambridge Isotope Laboratories, Inc.	
Deuterium dimethyl sulfoxide	Sigma Aldrich	99.9% atom D
Pyridine	Fischer Chemicals	Anhydrous, pre-mixed with molecular sieves 3A
Dimethyl sulfoxide	Fischer Chemicals	HPLC grade
Sodium carbonate	Fischer Chemicals	Anhydrous
Potassium Bromide	ACROS Organics	99.999%, (trace metal basis),
Chromium(III) acetylacetonate	Sigma Aldrich	99.99% Trace metals
N-hydroxy-5-norbornene-2,3-dicarboximide	Sigma Aldrich	97%
2-Chloro-4,4,5,5-tetramethyl-1,2,3-dioxaphospholane	Sigma Aldrich	95%

### 2.2 Analysis methods

#### 2.2.1 Nuclear magnetic resonance analysis

For ^1^H, ^31^P, and ^13^C NMR, the spectra were acquired using Bruker Avance NMR (300 MHz) at 25°C equipped with a BBO probe. HSQC NMR spectra were acquired using Bruker Avance Bruker AVANCE III (600 MHz) at 25°C equipped with a cryoprobe. The sample preparation and acquisition parameters has been explained in the specification table. Data analyzed methods: Topspin 3.6.1 software was used to process the obtained spectrum: Fourier transformation, baseline correction, and calibration. A semi-quantitative analysis of the ^31^P, ^13^C, ^1^H, and HSQC spectrum was performed based on previous works [12,13].

**Table 1:** CO_2_ evolution during the hydroxyethyl modification.

Sample	Temp./ °C	Time/h	CO_2_ (mL/g)	dCO_2_/dt (mL·g^−1^·h^−1^)	Sample	Temp.	Time/h	CO_2_ (mL/g)	dCO_2_/ dt(mL·g^−1^·h^−1^)
No. 2	80	0	0	0	No. 8	120	0	0	0
		0.25	5.2	13.6			0.5	30.8	61.6
		0.5	7.2	5.6			1	44	26.4
		0.75	9.2	4.8			1.5	66.8	45.6
		1	10.8	4			2	86.8	40.0
		1.5	13.6	3.6			2.5	90	6.4
		2	15.6	2.8			3	92	4.0
							3.5	94	4.0
No. 3	100	0	0				4	94	
		0.25	3.2	12.8					
		0.50	8	19.2	No. 9	120	0	0	33.6
		0.75	12	16			0.17	5.6	34.8
		1.08	16.4	13.2			0.33	11.6	33.6
		1.42	23.6	21.6			0.50	16.8	33.6
		2.00	32	14.4			0.67	22.8	36.0
							0.83	28.8	54.0
No. 4	100	0	0	17.9			1.00	40.8	62.4
		0.33	6	12.7			1.17	49.6	54.0
		0.58	9	8.0			1.33	58.8	51.6
		0.83	11	14.3			1.50	66.8	48.0
		1.08	15	23.1			1.72	77.2	39.9
		1.58	26	11.2			1.83	80.8	27.6
		4.08	54	17.9			2.00	84.8	22.3
							2.17	88.4	11.8
							2.33	88.8	7.1
No. 5	100	0	0				2.5	90.8	6.5
		0.25	9.6	38.4			2.83	91.2	1.8
		0.5	10.4	20.8			3.00	91.6	3.5
		0.75	14.8	10.4			3.17	92.4	3.6
		1	17.2	13.6			3.33	92.8	2.4
		1.5	23.6	11.2			4.00	94.4	1.8
		2	29.6	12.4			6.00	97.0	
		2.5	36.8	13.2					
		3	42	12.4	No. 10 Phloretic acid	120	0.00	0	360
		3.5	46.8	10			0.17	60	166
		4	50.8	8.8			0.67	143	141
		4.5	53.6	6.8			1.00	190	60
		5	56	5.2			1.33	210	30
		5.5	58.4	4.8			1.67	220	15
		6	60	4			2.00	225	
									
No. 6	100	0	0	17.6	No. 12 SKL-BC	120	0.00	0	144
		1	17.6	17.1			0.17	24	66.4
		1.58	27.6	24.0			0.67	57.2	56.4
		2.33	45.6	14.4			1.00	76	24
		2.75	51.6	13.9			1.33	84	12
		3.58	63.2	12.9			1.67	88	0
		4.67	77.2	0.6			2.00	88	
		5.92	78						
									
No. 7	120	0	0	72					
		0.25	18	64					
		0.5	34	81.6					
		0.75	54.4	102.4					
		1	80	16					
		1.25	84	4.8					
		1.5	85.2	3.2					
		1.75	86	0					
		2	86	0

**Table 2:** Quantitative ^31^P NMR analysis (mmol/g) and collected and theoretical CO_2_ amount during the modification.

No		Temp./°C	Time / h	CO_2_ / (mL/g)	AlOH^a^	C_5_ sub ArOH b	C_5_ free ArOH c	Total ArOH d	COOH e	DM/%^f^	Theoretical CO_2_ (mmol/g)
1	SKL^g^				2.71	2.29	2.54	4.83	0.68		
2		80	2	10	2.60	2.18	2.45	4.63	0.58	36	0.25
3		100	2	32	2.87	1.76	1.95	3.71	0.26	44	1.31
4		100	4	54	3.20	1.35	1.44	2.79	0.15	53	2.28
5		100	6	60	3.62	0.94	0.93	1.87	0.15	66	3.21
6		100	6	78	4.09	0.54	0.34	0.88	0.16	80	3.54
7		120	2	86	4.24	0.39	0.18	0.57	0.29	83	4.70
8		120	6	95	3.80	0.29	0.12	0.41	0.06	90	4.93
9		120	4	97	3.56	0.29	0.14	0.44	0.05	89	4.91
10	Phloretic acid	120		225							12.04
11	SKL-BC^h^				2.57	2.31	2.98	5.29	0.67		
12		120	2 - 3	88	4.72	0.37	0.16	0.52	0.05	88	4.73

a aliphatic hydroxyl groups (AlOH, 150 -146 ppm)

b ortho substituted aromatic hydroxyethyl groups (C5 sub, 144.5-141.5 ppm)

c ortho free aromatic hydroxy groups (C5 free, 141.5 – 136 ppm)

d Total aromatic hydroxyl groups (ArOH)

e carboxylic acid (COOH, 136 - 133 ppm)

f degree of modification (DM, AlOH/(ArOH+AlOH+COOH))

g softwood kraft lignin from LignoForce

h softwood kraft lignin (BioChoice)

**Table 3:** Semi-quantitative ^13^C NMR analysis of acetylated hydroxyethyl lignin during the modification (100Ar), the carbon signal from the aromatic region (155-100 ppm) was used as an internal standard.

No.	CO_2_ / (mL/g)	Pri-AlOH^a^	Sec-AlOH^b^	C_5_ subArOH^c^	C_5_ freeArOH^d^	Total OH	ArH^e^	Carbonate^f^	G^g^	MeO^h^	HE^i^
1	0	27.8	13.9	24.6	30.3	96.6	217.7	4.0	80.5	81.1	30
2	10	26.2	12.9	29.7	29	98.0	216.4	4.8	81.1	82.1	34
3	32	35.5	10.6	21.3	25.2	94.2	215.3	10.8	80.7	82.1	40
4	54	46.3	9.1	12.6	21.6	95.5	213.8	11.5	82.6	81.2	57
7	86	79.7	6.5	5.3	4.0	95.6	205.8	24.6	79.0	78.7	90

a primary aliphatic hydroxyl groups (Pri-AlOH, 171.5 – 169.7 ppm)

b secondary aliphatic hydroxyl groups (Sec-AlOH, 169.7 – 169.0 ppm)

c ortho free aromatic hydroxyethyl group (C5-sub, 169 -168.3 ppm)

d ortho substituted aromatic hydroxyethyl group (C5-free, 168.3 – 166.5 ppm)

e aromatic hydrogen (ArH, 125 -100 ppm)

f carbonate (157.4-153.7 ppm)

g guaiacyl (G, 115 – 106 ppm)

h methoxy (MeO, 58 -54 ppm)

i hydroxyethyl (∫_(75 - 58ppm)_)

**Table 4:** Molecular weight and PDI of different lignin resource during the modification; PSS was used as standard for the conventional calibration analysis (dRI).

No.	CO_2_(mL/g)	MW_dRI			MW_LS		
		M_w_/kDa	M_n_/kDa	PDI	M_w_/kDa	M_n_/kDa	PDI
1	0	9.2	0.7	12.2	28.3	7.7	3.7
2	10	19.4	0.9	22.3	41.9	17	2.5
3	32	22.7	0.8	28.8	97.1	24.6	3.9
4	54	21.5	0.66	32.8	119.5	19.3	6.2
7	86	116.6	0.8	141	278.5	50.94	5.5

#### 2.2.2 Gel permeation chromotography combined with MALS and dRI

PSS standard samples with molar masses of 1.1 kDa, 2.0 kDa, 4.29 kDa, 10 kDa, 29.5 kDa, 63.9 kDa, 145 kDa, and 470 kDa were prepared by dissolving 10 mg PSS in 1 mL DMSO at 50°C for 48 hrs until they were thoroughly dissolved in the solvent. Gel permeation chromatography (GPC) measurements were carried out using Agilent 1100 GPC equipment (USA) consisting of a pump, an autosampler, and a column oven set at 35°C. 20 μL lignin solution was injected into the system and separated. Two types of PolarGel column (PolarGel M and L, Agilent) were used to fractionate the lignin at 35°C using DMSO/LiBr (0.5% w/v) as eluent at 0.5 mL/min. The fractionated lignin was then analyzed by multi-angle laser light scattering (MALLS, Wyatt Tech. CA, USA), and the optilab T-rEX differential refractive index detector (dRI, Wyatt Tech. CA, USA). The data were collected and analyzed by ASTRA 6.0 software. The dn/dc value was calculated through the dRI traces by an on-line method using ASTRA software [14].

## Declaration of Competing Interest

The authors declare that they have no known competing financial interests influenced the work reported in this article.
